# Function Analysis of Heme Peroxidase Genes, MpPxd2 and MpPxd4, Under Thiacloprid Exposure in the Neonicotinoid-Resistant *Myzus persicae* (Sulzer)

**DOI:** 10.3390/antiox13121453

**Published:** 2024-11-27

**Authors:** Wenhua Rao, Feng Chen, Xianzhi Zhou, Jun Wang, Lei Lin, Guocheng Fan, Jinfeng Hu

**Affiliations:** Fujian Engineering Research Center for Green Pest Management, Key Laboratory for Monitoring and Integrated Management of Crop Pests, Institute of Plant Protection, Fujian Academy of Agricultural Sciences, Fuzhou 350002, China

**Keywords:** *Myzus persicae*, antioxidant defense, heme peroxidase, thiacloprid exposure, neonicotinoid resistance

## Abstract

The green peach aphid, *Myzus persicae*, is a notorious pest worldwide. We collected a field population of the pest (FZQ-F) that exhibited high resistance to neonicotinoids. Exposure to neonicotinoids can induce oxidative damage in animals; however, it remains unclear whether antioxidant enzymes contribute to the innate immune response of neonicotinoid-resistant pests against high doses of insecticides. Treatment with sublethal doses of thiacloprid (LC_10_ and LC_25_) for 3, 6, 12, 24, 48, and 72 h resulted in significantly increased reactive oxygen species (ROS), including H_2_O_2_ content, in FZQ-F adults, indicating insecticide-induced oxidative stress. Additionally, the peroxidase activity in FZQ-F adults increased after thiacloprid exposure. Using comparative genomics, we identified 31 heme peroxidases in *M. persicae* with a typical “2Cys” structure, and phylogenetic analyses divided them into five groups. Comparative transcriptomes revealed that MpPxd2 and MpPxd4 were significantly upregulated in thiacloprid-treated aphids. Thiacloprid exposure significantly induced MpPxd2 and MpPxd4 expression levels, consistent with high H_2_O_2_ content and peroxidase activity. The knockdown of MpPxd2 or MpPxd4 in FZQ-F increased their susceptibility to imidacloprid, thiacloprid, and thiamethoxam, verifying the protective role of the heme peroxidases against neonicotinoids in aphids. The knockdown of MpPxd2 or MpPxd4 also led to shorter longevity and a low fecundity of adult aphids at 31 °C compared to controls. The results show that MpPxd2 or MpPxd4 is important in how cells respond to oxidative stress and may help resistant *M. persicae* pests to handle neonicotinoids.

## 1. Introduction

The green peach aphid, *Myzus persicae* (Sulzer) (Hemiptera: Aphididae), is well known as one of the most destructive aphids in agriculture, causing serious economic losses worldwide through the transmission of aphid-borne viruses. Currently, chemical control remains the primary method for managing peach aphids [1]. The extensive use of chemical insecticides has unavoidably caused the development of insecticide resistance worldwide, resulting in the failure to control *M. persicae* with chemicals. Neonicotinoids, such as imidacloprid, thiacloprid, and thiamethoxam, belong to a class of insecticides that specifically target the nicotinic acetylcholine receptors (nAChRs) in insects. For over thirty years, farmers have been using them as the primary insecticide products to control *M. persicae* in fields. Neonicotinoid resistance in *M. persicae* communities has also been detected at high levels in populations from Greece, Australia, China, etc. [2,3,4]. Understanding the resistance mechanisms of peach aphids against neonicotinoid insecticides is vital for developing effective resistance management strategies and guiding the responsible use of these chemicals.

Recently, more and more studies have focused on key effector molecules that regulate the constitutive overexpression of different genes responsible for insecticide resistance [5]. According to previous research, insects have developed a way to deal with xenobiotic stress by using reactive oxygen species (ROS) as transcription factors to make more enzymes and transporters that make them resistant to pesticides [6,7]. ROS, such as hydrogen peroxide (H_2_O_2_), hydroxyl radicals (-OH), and superoxide anions (O_2_^·−^), are involved in a multitude of biological processes, including cell signaling and immune responses within organisms. All pesticides cause oxidative stress in animals due to an asymmetry between the excessive generation of ROS and the antioxidant defense systems’ ability to neutralize them [8]. Insecticide resistance genes, such as the ABC transporter that makes the red mite *Dermanyssus gallinae* resistant to beta-cypermethrin [9], are overexpressed when ROS bursts take place. Meanwhile, the enhanced production of ROS results in oxidative stress, leading to DNA damage and genotoxicity in animals. The antioxidant defense system is important for all organisms to mitigate the induction of ROS [10]. Peroxidases (PODs) are a key component of this defense system, helping to detoxify reactive intermediates and protect cells from oxidative damage.

Insect peroxidases, a critical subset of the peroxidase family, are multi-domain proteins with iron porphyrins as their prosthetic group. Despite the discovery of peroxidase activities in various insects over three decades ago [11,12], they remain less studied compared to their mammalian counterparts. Researchers have performed comparative genomics and phylogenetic analyses on several insects, including *Bombyx mori*, *Aedes aegypti*, and *Apis mellifera*, in order to understand the evolutionary relationships between peroxidase genes in different insect species [13,14,15]. Studies have revealed that insects possess a variety of peroxidases, including heme peroxidase (Pxd), glutathione peroxidase (Gpx), thioredoxin (Trx)-dependent peroxidase (Tpx), and thioredoxin/glutaredoxin (Trx/Grx). Insect heme peroxidases are a significant group of peroxidases with a variety of functions. For instance, the heme peroxidase gene Cysu in *Drosophila melanogaster* is associated with wing formation. Another heme peroxidase, chorion peroxidase, is critical for eggshell formation in insects such as *D. melanogaster* and *A. aegypti* [16,17].

Scavenging ROS and shielding cells from oxidative damage caused by xenobiotic stimulation are among POD’s most crucial roles in insects. Research has shown that certain insecticides can improve POD activity in insects. Six different insecticides, including cypermethrin, avermectin, emamectin benzoate, chlorantraniliprole, and deltamethrin, can induce POD activities in *Helicoverpa assulta* [18]. Exposure to deltamethrin also aroused POD activity in *Pieris rapae* [19]. Transcript analysis proved the overexpression of some POD genes in insect responses to insecticide exposure. Vontas and Small [20] found that glutathione peroxidase plays an important role in pyrethroid resistance in brown planthoppers. *Grapholita molesta* increased its expression levels of GmTrx2 and GmTrx-like1 to clean up extra ROS induced by environmental stresses such as low or high temperatures, H_2_O_2_, and emamectin benzoate [21]. Additionally, the thioredoxin peroxidase PrTPX1 aids *P. rapae* in managing the oxidative stresses resulting from exposure to chlorantraniliprole [22].

Some studies have documented the effects of neonicotinoids on ROS and PODs in animals. When animals such as the Eurasian carp *Cyprinus carpio* [23] and pollinators [24] are exposed to neonicotinoids, they may cause DNA damage, apoptosis, protein oxidation, and lipid peroxidation [25]. However, the effects of neonicotinoids on the PODs of pests have also been widely reported. Researchers have detected improved POD activity in NEO-resistant *M. persicae* [4,26]. Importantly, neonicotinoid-resistant pests, such as thiamethoxam-resistant *Bemisia tabaci* and field-collected neonicotinoid-resistant *M. persicae*, showed an upregulation of some POD genes [4,27]. These studies suggest that there may be a link between this enzyme and the fact that *M. persicae* is resistant to neonicotinoids. Thiacloprid is an important neonicotinoid because it is much more efficient against field-resistant *M. persicae* than imidacloprid [4], as well as being much less acutely toxic to pollinators than other neonicotinoids [28]. To find out what role heme POD plays in making *M. persicae* resistant to neonicotinoids, we gathered a field population of *M. persicae* that was resistant to neonicotinoids, in order to look for possible resistance-related heme POD genes. First, we systematically analyzed and compared the heme PODs of *M. persicae* with those of *Acyrthosiphon pisum* (Hemiptera: Aphididae) and *Bemisia tabaci* (Hemiptera: Aleyrodidae), two other important agricultural pests. We investigated the transcriptomic profile function of resistance-related heme POD genes in neonicotinoid-resistant aphids and susceptible aphids. We also looked at how thiacloprid affected the differentially expressed peroxidase genes and their function, which may provide much information for neonicotinoid-resistance management in the aphid.

## 2. Materials and Methods

### 2.1. Insects

The laboratory population of *M. persicae* (FFJ-S) was a susceptible strain that is vulnerable to insecticides [4]. The field population (FZQ-F) that exhibited resistance to neonicotinoids was collected from cabbage fields in Fuzhou on 19 January 2024 (23°46′28′′ N, 113°49′59′′ E). The *M. persicae* strains were cultivated on *Brassica campestris* ssp. Chinensis (L.) seedlings under conditions of 21 ± 2 °C and 65 ± 5% relative humidity in a light/dark cycle (LD, 16:8) at the Fujian Institute of Plant Protection, Fujian Academy of Agricultural Sciences, Fuzhou. The field strain was maintained for two generations prior to the related experiment.

### 2.2. Insecticides

The three insecticides, thiacloprid (97.5% purity; Bayer AG, Leverkusen, Germany), thiamethoxam (98% purity; Syngenta Group, Dielsdorf, Switzerland), and imidacloprid (97% purity; Bayer AG, Leverkusen, Germany), were selected for the bioassays.

### 2.3. Toxicity Test Methods

The leaf-dip method, as recommended by the Insecticide Resistance Action Committee (IRAC) in 2023, was employed to assess the toxicity of the three insecticides, as described by Hu et al. [4]. This involved preparing five to seven different concentrations of each insecticide, using 0.1% Triton-100 as a solvent, with four replicates for each concentration. Clean leaf discs were cut from *Brassica oleracea* L. plants using a metal tube and then immersed in the test solution for a duration of 10 s. After air-drying, these leaf discs were placed on a 1% agar plate with a depth of 20 mm, within a petri dish measuring 30 mm in diameter and 40 mm in depth. Following this, twenty apterous (wingless) adult aphids were carefully transferred onto each leaf-disc using a paintbrush. Each setup was subsequently covered with a snug-fitting, aerated lid. The mortality of the aphids was evaluated after three days. Aphids were considered to be dead if they were unable to right themselves within 10 s after being placed on their backs. The LC_10_, LC_25_, and LC_50_ doses were determined by means of probit analyses. Four replicates were conducted.

### 2.4. Preliminary Search and Identification of Heme Peroxidases in the M. persicae Genome

The initial search for members of the *M. persicae* peroxidase gene family (MpPxds) employed three methods: keyword search, HMM (hidden Markov model), and BLAST. In order to learn about the polygenetic relationships with other insects, we also searched heme peroxidase genes in two other Hemipteran insects: the tobacco whitefly *Bemisia tabaci* and the pea aphid *Acyrthosiphon pisum*. Heme peroxidase genes for *M. persicae*, *B. tabaci*, and *A. pisum* were abbreviated as “MpPxd”, “BtPxd”, and “AcPxd”, respectively. The preliminary data for MpPxds members were collected using the following steps: (1) performing a keyword search for “heme peroxidase” in the *Myzus persicae* database (https://bipaa.genouest.org/sp/myzus_persicae_g006, accessed on 18 February 2024); (2) downloading the heme peroxidase protein-conserved domain model (PF03098) from the Pfam library (http://pfam.xfam.org/, accessed on 19 February 2024) using HMMER 3.4_Windows software (http://hmmer.org/, accessed on 19 February 2024) [29]; and (3) accessing the *M. persicae* database (https://bipaa.genouest.org/sp/myzus_persicae_g006, accessed on 19 February 2024) to retrieve 31 peroxidase gene protein sequences, followed by the application of the BLASTP method and the elimination and combination of duplicated genes. Similar steps were also followed in the search for the peroxidase genes of *B. tabaci* (https://www.ncbi.nlm.nih.gov/datasets/genome/GCF_001854935.1/, accessed on 2 October 2024) and *A. pisum* (https://bipaa.genouest.org/sp/acyrthosiphon_pisum/, 2 October 2024).

### 2.5. Construction of the Phylogenetic Tree of Heme Peroxidase for M. persicae, B. tabaci, and A. psium

The multiple sequence alignments of the peroxidase proteins for all three insects—*M. persicae*, *B. tabaci*, and *A. pisum*—and *M. persicae* alone were analyzed via MEGA 11.0 software utilizing the Muscle algorithm. Then, the neighbor-joining (NJ) method was employed, along with 1000 bootstraps, to construct the evolutionary tree. The remaining parameters were set to their default levels [30,31].

### 2.6. MpPxd Structure and Protein Domain Analysis

Subsequently, for these obtained MpPxd genes, the conserved motifs were examined according to the online MEME Suite (http://meme-suite.org/tools/meme, accessed on 22 February 2024), and conservative projections of protein domains were retrieved through the Conserved Domains Database (https://www.ncbi.nlm.nih.gov/Structure/cdd/wrpsb.cgi, accessed on 22 February 2024) using TBtools software v2.136 [32]. The complete gene structure information for the MpPxds, including gene length, coding sequence (CDS) position, and gene functional domain prediction, was obtained using the “Gene Structure View” module of TBtools.

### 2.7. Treatment of FZQ-F Apterous Adult Aphids by Sublethal Doses of Thiacloprid

Apterous adult FZQ-F aphids were treated with thiacloprid at doses of LC_10_ and LC_25_ according to the toxicity test. The strains FFJ-S and FZQ-F, treated only with 0.01% Triton X-100 (Sigma-Aldrich, Saint Louis, MI, USA), were used as the controls. In order to select upregulated MpPxds, samples collected 24 h after thiacloprid treatment, with untreated FFJ-S and FZQ-F populations together, were used for transcriptome studies. Meanwhile, for expression induction studies, FZQ-F apterous adults were treated with LC_10_ and LC_25_ doses of thiacloprid for 3, 6, 12, 24, 36, 48, and 72 h to determine the effects of thiacloprid on their peroxidase activity, H_2_O_2_ content, and expression of significantly upregulated MpPxd genes. The experiment comprised three replicates, each consisting of 30 adults. The insects were flash-frozen in liquid nitrogen and maintained at −80 °C.

### 2.8. Measurement of ROS Levels

*Myzus persicae* FQZ-F were treated as described in Section 2.6. We carefully removed the intestines of *M. persicae* using forceps under a dissecting microscope after a 24 h and 72 h period. These intestines were then transferred into a PBS solution containing 10 μM DCFH-DA stain (provided by Beyotime, Shanghai, China). The staining process involved incubating the intestines in a 37 °C incubator, shielded from light, for 20 min. We gently agitated the sample every 5 min during this time. For the positive control, 10 μM Rosup (provided by Beyotime, Shanghai, China) was added to stimulate reactive oxygen species (ROS) production, with the treatment continuing for an additional 20 min. We carefully removed the staining solution using filter paper after the incubation and replaced it with a PBS suspension for observation using a laser confocal microscope (excitation at 488 nm, emission at 525 nm) (LSM 900, Zeiss, Göttingen, Germany) [33,34].

### 2.9. Determination of Protective Enzyme Activities, Hydrogen Peroxide Content (H_2_O_2_), and Protein in M. persicae

The peroxidase activities, hydrogen peroxide content, and protein were measured using peroxidase assay kits (catalog no. A084-3-1, Guaiac method), hydrogen peroxide assay kits (catalog no. A064-1-1, colorimetry), and total protein quantitative assay kits (A045-2, Bradford method) from the Nanjing Jiancheng Bioengineering Institute (Nanjing, China), respectively, following the manufacturer’s protocols. For these experiments, 30 apterous adult aphids were homogenized and five replicates were conducted.

### 2.10. The MpPxd Transcriptome Profiles Under Various Treatments

The total RNA from ~600 apterous adult aphids per treatment (1800 aphids for three replicates) was isolated using the TRIzol™ reagent (Invitrogen, Carlsbad, CA, USA) in accordance with the manufacturer’s instructions. RNA purification, reverse transcription, library construction, and sequencing were carried out at Shanghai Majorbio Bio-pharm Biotechnology Co., Ltd. (Shanghai, China) following the manufacturer’s protocols. The sequencing library was constructed on the NovaSeq X Plus platform (PE150) using the NovaSeq Reagent Kit. We compared the transcriptome profiles of MpPxds between different treatments.

To identify the differential expression of MpPxds between untreated FFJ-S, FZQ-F, and LC_10_- or LC_25_-thiacloprid-treated FZQ-F, the expression level of each transcript was quantified using the FPKM (fragments per kilobase of transcript per million mapped reads) metric. We employed RSEM for gene abundance quantification. For differential expression analysis, we utilized DESeq2 or DEGseq [35,36]. Genes with |log2FoldChange| ≥ 1 and FDR < 0.05 (DESeq2) or FDR < 0.001 (DEGseq) were deemed significantly differentially expressed.

### 2.11. Expression Induction of Selected MpPxds via Thiacloprid Exposure

The mRNA levels of two selected MpPxd genes (MpPxd2 and MpPxd4) in treated aphids, as described in Section 2.6, were measured via RT-qPCR using SYBR^®^ Green Supermix (Thermo Fisher, Waltham, MA, USA) in a qTOWER 2.2 real-time quantitative PCR system (Analytikjena, Jena, Germany). We isolated total RNA as previously outlined and measured its concentration using a ScanDrop 100 spectrophotometer (Analytikjena, Jena, Germany), adhering to the manufacturer’s guidelines. The RNA was diluted to 0.8 μg/μL with diethyl pyrocarbonate (DEPC)-treated H_2_O, and 0.8 μg of RNA was reverse-transcribed in a 20 μL reaction volume using the TUREscript 1st Strand cDNA Synthesis Kit (Aidlab, Beijing, China), with the actin gene serving as an internal control (NCBI gene ID: 836110). Each RT-qPCR was performed in a 20 μL mixture consisting of 1 μL of sample cDNA, 1 μL of each primer (200 nM), 6 μL of DEPC-treated H_2_O, and 10 μL of 2×SYBR^®^ Green Supermix. The qPCR cycling parameters were: 95 °C for 3 min, followed by 39 cycles of 95 °C for 10 s and 58 °C for 30 s. The analysis was conducted using a plate reader. Melting curve analysis was carried out from 60 to 95 °C. The primers for these genes were designed using Primer Express 3.0 software based on the target gene sequences from NCBI, and they are listed in Supplementary Table S1.

### 2.12. RNA Interference

MpPxd2 and MpPxd4 double-stranded RNAs (dsRNAs) were produced in accordance with the manufacturer’s instructions using the T7 high-yield transcription kit (Invitrogen, USA). The primers utilized for dsRNA synthesis are listed in Supplementary Table S1. A total of 150 ng/μL of the dsRNA that targeted the desired gene was added to the artificial diet, which was composed of a 0.5 M sterile sucrose solution. Artificial food containing dsRNA-EGFP was employed as a control. First, one end of the glass tube (2.5 cm in diameter and 3 cm in height) was sealed with a sealing film, then the artificial feed was added onto the sealing film, quickly covering it with another layer of paraffin film. After that, aphids were transferred to the glass tube for raising [4]. There were three biological replicates used in the experiment, and each included thirty aphids. RT-qPCR was used to assess the efficacy of the dsRNA in suppressing the expression of the two MpPxd genes after 48 h.

We treated brachypterous adults with the LC_50_ doses of these three neonicotinoids 48 h later to determine the susceptibility of *M. persicae* to thiacloprid, imidacloprid, and thiamethoxam after the RNAi of the MpPxd2 and MpPxd4. Adults treated with DEPC-water and dsRNA-GFP were used as the controls. The mortality of *M. persicae* was assessed after 48 h. Additionally, we examined the longevity of adults and fecundity in each female at 21 °C and 31 °C following RNAi treatment for the two genes. We fed the 4th-instar larvae with artificial food for 48 h to measure adult longevity and fecundity, and then we transported them to cabbage seedlings to calculate the days and offspring production until the aphids were dead. Each treatment for the mortality tests was replicated five times, and ninety treated apterous adults were used to calculate adult longevity and fecundity.

### 2.13. Western Blot Analysis

Protein extraction was performed from 500 adult apterous female adults per sample using the cell lysis buffer for the Western and IP Kit (Macklin) according to the manufacturer’s instructions. The protein concentration was assessed using the BCA Protein Assay Kit (Glpbio, Montclair, CA, USA), and 20 μg of total protein from each sample was analyzed (CWBio, Taizhou, China). Rabbit polyclonal antibodies against MpPxd2 and MpPxd4 were generated using synthetic peptides (Jiaxuan Biotech, Jiaozuo, China). The peptide sequence for MpPxd2 was T-L-K-R-K-C-D-K-E-F-H-L-D-F-K (amino acids 663 to 677), and the peptide sequence for MpPxd4 was Y-C-P-L-R-K-R-K-G-S-L-V-C-D-G (amino acids 254 to 268). The specificity of the antibodies was verified by a BLAST search of the peptide sequences against the genome of *M. persicae* (https://bipaa.genouest.org/sp/myzus_persicae_g006/, accessed on 22 February 2024). Western blots were probed with the GAPDH antibody (Abcam, Shanghai, China), which served as a loading control.

For Western blot analysis, aphid samples treated with DEPC, dsRNA-GFP, dsRNA-MpPxd2, and dsRNA-MpPxd4 were homogenized in Tris buffered saline (TBS), respectively. The homogenates were centrifuged at 12,500× *g* for 10 min to obtain soluble proteins. A 12% SDS-PAGE denaturing gel electrophoresis was conducted on a JY-SCZ4+ miniprotean apparatus (Junyi, Beijing, China). Equal protein amounts, quantified by the DC Protein Assay Kit II (Bio-Rad), were mixed with SDS sample loading buffer, boiled, and electrophoresed under denaturing conditions. Proteins were then electroblotted onto a PVDF membrane (Millipore, St. Charles, MI, USA) using a JY-ZY3 Semi-dry transfer blotter (Junyi). After incubation with anti-His·tag rabbit antiserum (1:1000; Beyotime) and horse-radish peroxidase-conjugated goat anti-rabbit IgG (1:4000; Beyotime), MpPxd2 and MpPxd4 were detected with rabbit polyclonal antibodies (1:1000 in TBS) and visualized using the Western Blotting Luminol reagent (Bio-Rad, Santa Rosa, CA, USA).

### 2.14. Data Analysis

The data on H_2_O_2_ content, enzymatic activities, gene expression, adult longevity, and fecundity across different treatments, as well as the mortality of RNAi-treated aphids exposed to insecticides versus the control group, were analyzed using the Student’s *t*-test and a one-way analysis of variance (ANOVA), followed by Tukey’s multiple comparison test. These analyses were conducted using the DPS Data Processing System (developed by Hangzhou Ruifeng Technology Ltd., Zhejiang, China), as described by Tang and Zhang [37]. The results are shown as mean ± SE. Statistical significance was determined at *p* < 0.05, 0.01, 0.001, or 0.0001.

The LC_10_, LC_25_, and LC_50_ values for each insecticide against adult apterous aphids were calculated using the DPS Data Processing System. The LC_50_ values were considered significantly different if their 95% confidence intervals (CIs) did not overlap. The resistance factor (RF) was calculated as the ratio of the LC_50_ value of the field population to that of the FFJ-S strain. Resistance levels were classified based on the World Health Organization’s (WHO) 1980 standards: susceptible (RF < 3), decreased susceptibility (RF = 3–5), low resistance (RF = 5–10), moderate resistance (RF = 10–40), high resistance (RF = 40–160), and extremely high resistance (RF > 160).

## 3. Results

### 3.1. Neonicotinoid Resistance in FZQ-F

The FZQ-F strain showed high resistance to thiacloprid (RF = 44.1-fold; LC_50_ = 28.62 mg/L) and imidacloprid (RF = 93.3-fold; LC_50_ = 43.1 mg/L), along with moderate resistance to thiamethoxam (RF = 17.5-fold; LC_50_ = 19.82 mg/L). The LC_10_ and LC_25_ values of thiacloprid in FZQ-Fwere 11.77 mg/L, 28.62 mg/L, and 125.908 mg/L, which were used as doses for sublethal treatment in the following experiment (Table 1). The LC_50_ doses of the three neonicotinoids were used to determine the toxicity effects of RNA interference (RNAi).

### 3.2. Identification and Characterization of MpPxd Gene Family Members

In this study, 45 candidate peroxidase genes were identified through keyword search, 58 through HMMER search, and 53 through BLASTP search. After further deduplication, motif analysis, and functional domain analysis, 32 candidate peroxidase genes were finally selected. The genes inferred using HMMER (PF03098) were characterized by conserved and stable structures, while the searched genes included two other types of results.

The 31 peroxidase genes are presented in Table 1. The CDS region lengths ranged from 1667 to 5276 bp, and the number of exons varied from 7 to 26. These 31 Pxd genes of *M. persicae* had amino acid sequences ranging from 538 (MpPxd7) to 1351 (MpPxd25), molecular weights of 61.94~153.4 kD, isoelectric points of 4.89~8.47, and signal peptide lengths of 17~26 amino acids. The peroxidase gene secondary structures had α-helix compositions ranging from 16.35% to 32.34%, β-sheet compositions ranging from 12.14% to 23.4%, and random coil compositions ranging from 47.92% to 67.15%. Their hydrophilic coefficients ranged from around −0.545 to −0.121, indicating that the Pxd family proteins were all hydrophilic proteins, which was consistent with the fact that the Pxd family proteins were soluble proteins. The specific information for the corresponding numbering, gene start and end locations, CDS length, number of exons, and encoded protein characteristics of each gene are displayed in Table S2.

### 3.3. MpPxd Evolutionary Tree, Protein Sequence Comparison, Gene Structure, and Conserved Structure

**Figure 1:**
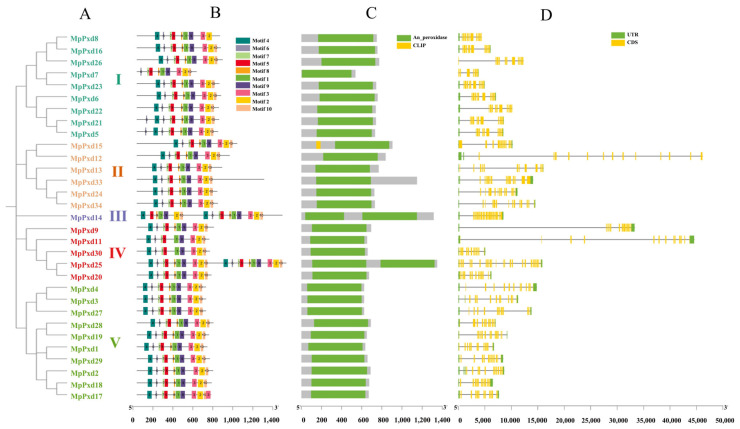
Analysis of M. persicae MpPxds: (**A**) Intraspecific phylogenetic tree of MpPxd members. (**B**) Distribution of conserved motifs in MpPxd proteins. (**C**) Conserved domains of MpPxd proteins. (**D**) Intron–exon structure and functional domains of the conserved structure of MpPxds.

The phylogenetic analysis of MpPxds revealed their classification into five distinct groups, as depicted in Figure 1A. Group V was the most numerous, with ten genes, followed by Group I with nine, Group II with six, Group IV with five, and Group III with just one gene. Within each group, the genes exhibited higher levels of similarity in terms of gene structure, amino acid ratio, conserved motif elements, and functional domain characteristics. All MpPxd heme peroxidases contained at least 10 motifs, with MpPxd14 and MpPxd25 having 17 and 20 motifs (Figure 1B), respectively. Most genes within the five groups possessed a single peroxidase domain (Figure 1C). However, MpPxd14 and MpPxd23 were unique in having two peroxidase domains: one connected to a transmembrane domain (TM) at the amino terminus (DBLPX-N) and the other associated with the von Willebrand type C (VWC) domain at the carboxyl terminus (DBLPX-C). These proteins shared a structurally linked DBLPX subfamily domain, which was consistent across other insects. Additionally, the C-terminus of Group I, IV, and V genes also featured the VWC domain. MpPxd5 was notable for its possession of two conserved domains: the CLIP domain and the peroxidase domain. The CLIP domain is recognized as a regulatory domain that governs the proteinase activity of various trypsin family proteins, including Easter and pap2. After the cleavage of a conserved residue that maintains the protein in its zymogen form, the CLIP domain remains attached to the protease domain.

Coding sequences (CDSs) are important for functional gene analysis, whereas untranslated regions (UTRs) contain important regulatory motifs such as transcription binding sites and miRNA targeting sites, which are important features of gene evolution. Figure 1D illustrates significant differences in the number of exons among MpPxds. The number of exons in the 31 MpPxds ranged from seven to twenty-six, among which nine genes in Group I had a lower number of exons—with only seven to eleven—than the other four genes. As the only gene in Group III, MpPxd14 had a total of 20 exons. The gene MpPxd25 in Group V contained the highest number of exons (26).

Multiple sequence alignments of the 31 MpPxds showed one disulfide bond at the N-terminus and the C-terminus, composed of four cysteine sites, confirming that MpPxds belong to the “2-Cys” peroxidase family (Figure S1). These MpPxds also contain five Ca^2+^-binding sites and one transition state stabilizer site at the N-terminus, as well as one heme-binding site and one iron-ion-binding site at the C-terminus. These findings are consistent with previous research and the analysis of heme peroxidase genes.

### 3.4. Phylogenetic Analysis of Deduced Amino Acid Sequences of MpPxds with Other Insects

**Figure 2:**
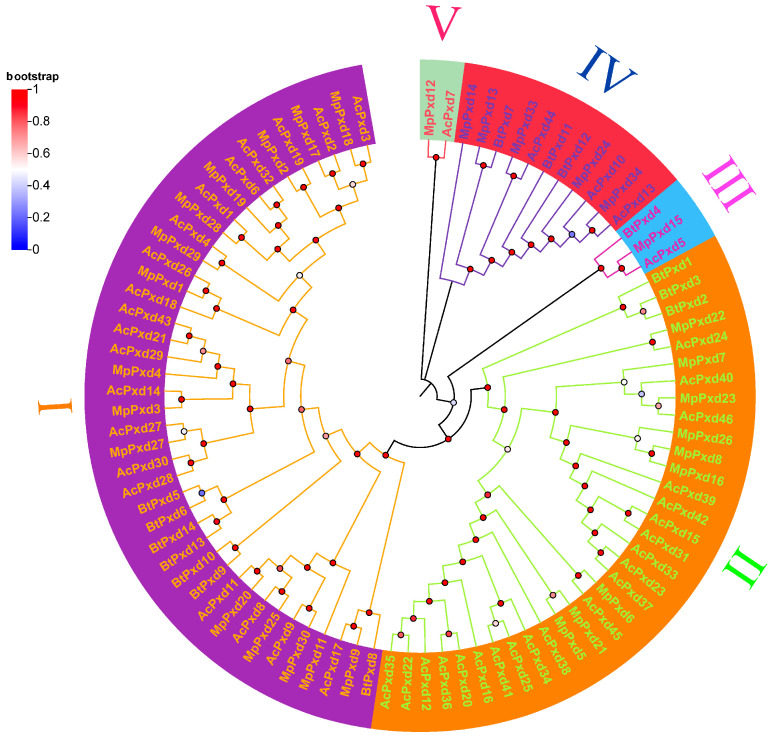
Phylogenetic analysis of heme peroxidases in *Myzus persicae* (MpPxds): “The phylogenetic tree was generated using ClustalW alignment of the nucleotide-binding domains (NBDs) from heme peroxidases of *A. pisum*, and *B. tabaci*. The bootstrap values at branch points were obtained from 1000 replications with MEGA 11.0, employing the maximum-likelihood method. The hollow diamond and solid circle denote distinct heme peroxidase proteins from *M. persicae*, *A. pisum*, and *B. tabaci*”. Roman numerals I–V with different colors indicate the different groups of heme peroxidases.

Through the genomes of *A. pisum* and *B. tabaci*, 46 and 14 heme peroxidases were confirmed, respectively, both fewer than the heme peroxidases in *M. persicae*. Phylogenetic analysis (Figure 2, Table S2) showed that the heme peroxidases in *M. persicae* form different monophyletic clades. These peroxidases all have the most conserved peroxidase domain (PF03098) in the peroxidase family. The phylogenetic tree study of heme peroxidase in the three Hemipteran insects (Figure 2) showed that all of the heme peroxidase genes were divided into five groups. The largest number of genes is found in Group I, which contains 42 genes, followed by Group II, which contains 33 genes.

### 3.5. Induction of H_2_O_2_ Content and PODs by Thiacloprid in FZQ-F

**Figure 3:**
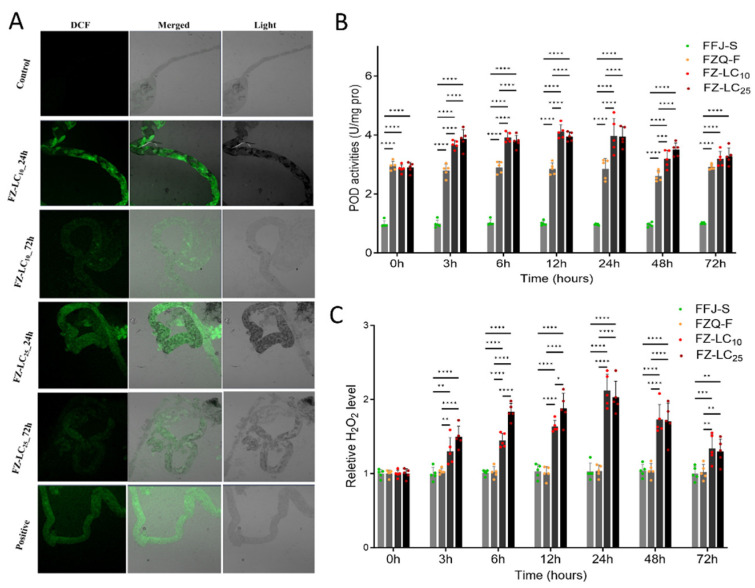
(**A**) Effects of thiacloprid on intestinal reactive oxygen species accumulation in the midgut of FZQ-F aphids. (**B**) Effects of thiacloprid on the activities of PODs in FZQ-F aphids. (**C**) Effects of thiacloprid on the H_2_O_2_ content in FZQ-F aphids. The significant differences are marked by asterisks: * at the 0.05 level; ** at the 0.01 level; *** at the 0.001 level; and **** at the 0.0001 level.

We focused on the generation of ROS in the intestines of *M. persicae* after exposure to thiacloprid (Figure 3A). In the positive group, we observed ROS signals throughout the intestine. We did not observe a clear ROS signal in the control group, but in the thiacloprid-treated group, we observed the formation of ROS and its distribution throughout the intestine. At both the LC_10_ and LC_25_ doses, we observed significantly more ROS after 24 h of exposure than after 72 h. Otherwise, thiacloprid exposure caused a significant increase in H_2_O_2_ content in the FZQ-F. The significantly higher H_2_O_2_ content persisted until the 72nd hour of thiacloprid exposure. We observed the highest values of H_2_O_2_ content 24 h after exposure (Figure 3C).

The findings indicated that the FZQ-F strain exhibited a significantly higher POD activity (3.01-fold) than the FFJ-S strain, as depicted in Figure 3B. In FZQ-F, thiacloprid had a significant effect on POD activity. We observed a significant increase in POD activity in FFJ-S at 3 h, 6 h, 12 h, 24 h, and 48 h after exposure to LC_10_ and LC_25_ doses of thiacloprid. However, after 72 h of exposure, the enzyme activity was similar to the control, and it returned to the control level after two weeks.

Using fluorescent probes, the experiment showed that the intestines of FZQ-F aphids that were treated with thiacloprid at LC_10_ and LC_25_ concentrations had a lot of reactive oxygen species (ROS) (Figure 3A). We found significantly higher ROS levels in the intestines 24 h post-treatment compared to the 72 h treatment. Furthermore, thiacloprid exposure significantly elevated the hydrogen peroxide (H_2_O_2_) content in the FZQ-F strain. The FZQ-F strain sustained this significant increase in H_2_O_2_ levels for up to 72 h after thiacloprid exposure. We observed the peak H_2_O_2_ content 24 h after thiacloprid exposure (Figure 3C).

### 3.6. The Expression Patterns and Transcriptional Responses of MpPxds to Thiacloprid

We analyzed the expression of 28 MpPxd genes in different strains of mature apterous aphids with RPKM > 0. There were six heme peroxidase genes that were highly expressed in both FFJ-S and FZQ-F: MpPxd9, MpPxd33, MpPxd28, MpPxd22, MpPxd24, and MpPxd11. FZQ-F had higher levels of eleven genes compared to FFJ-S: MpPxd11, MpPxd14, MpPxd2, MpPxd21, MpPxd24, MpPxd30, MpPxd33, MpPxd34, MpPxd4, MpPxd5, and MpPxd9.

**Figure 4:**
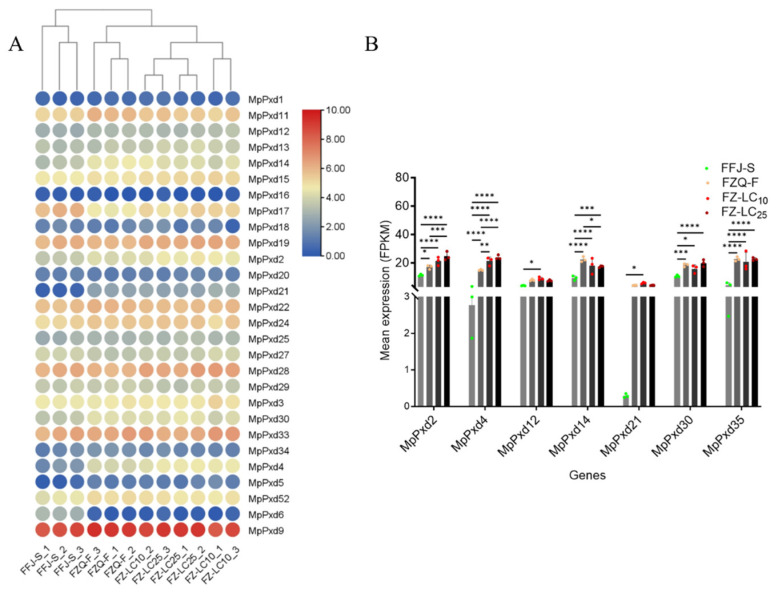
(**A**) Expression profiles of MpPxds in different aphid strains. The mRNA levels, depicted as normalized log2 (FPKM + 1) values, are presented in a gradient heatmap, where colors transition from blue (indicating low expression) to red (indicating high expression). (**B**) Mean expression of selected MpPxds in different aphid strains. The mRNA expression is represented by FPKM. FFJ-S represents susceptible aphids, while FZQ-F represents resistant field aphids. FZ-LC_10_ represents FZQ-F aphids that were exposed to LC_10_ dose thiacloprid for 24 h. FZ-LC_25_ represents FZQ-F aphids that were exposed to LC_25_ dose thiacloprid for 24 h. The significant differences are marked by asterisks: * at the 0.05 level; ** at the 0.01 level; *** at the 0.001 level; and **** at the 0.0001 level.

We also used the transcriptome profiles of FZQ-F female apterous adults treated with LC_10_ and LC_25_ doses of thiacloprid for 24 h, in order to evaluate the relative expression of MpPxds in response to thiacloprid exposure (Figure 4A). The comparative transcriptome analysis revealed an upregulation of seven MpPxd genes in FZQ-F and thiacloprid-treated FZQ-F compared to FFJ-S. To learn more about the differences between FZQ-F and thiacloprid-treated FZQ-F, we looked at seven genes. Only MpPxd2 and MpPxd4 showed significant overexpression in treated FZQ-F; they were also significantly overexpressed in FZQ-F when exposed to the LC_10_ or LC_25_ of thiacloprid after 24 h. This suggests that these two MpPxds may be involved in a protective response against thiacloprid.

### 3.7. Induction of H_2_O_2_ Content, POD Activities, and Expression of MpPxd2 and MpPxd4 in FZQ-F by the LC_50_ Dose of Thiacloprid

**Figure 5:**
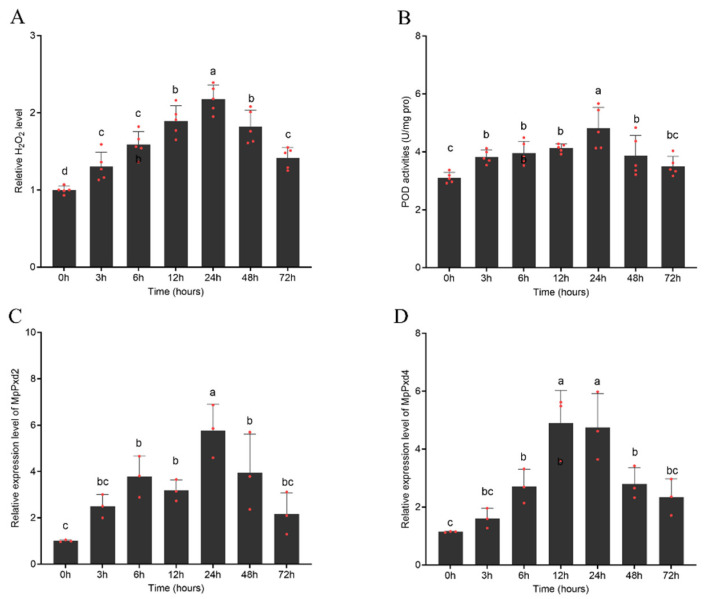
Induction of (**A**) H_2_O_2_ content and (**B**) POD activities, and expression of (**C**) MpPxd2 and (**D**) MpPxd4 in FZQ-F after the LC_50_ dose of thiacloprid. The expression levels were normalized to the β-actin gene. The bars marked with lowercase letters (a–c) indicate significant differences as determined by one-way ANOVA followed by Tukey’s multiple comparison test (*p* < 0.05).

We conducted analyses of the H_2_O_2_ content and POD activity in FZQ-F after exposing them to the LC_50_ dose of thiacloprid at different intervals. The LC_50_ dose of thiacloprid had a significant effect on the H_2_O_2_ content and POD activity in FZQ-F, as did time. At all tested exposure times, the H_2_O_2_ content was significantly higher in treated aphids than in untreated aphids. The H_2_O_2_ content increased from 3 h to 24 h, reaching its highest value after 24 h of exposure (Figure 5A). We also observed a significant increase in POD activity. We observed the highest values of POD activity at 24 h of exposure. The increased level of POD activity persisted until the 48th hour of the experiment and returned to the control level after 72 h of exposure (Figure 1A).

We also evaluated the induced expression of MpPxd2 and MpPxd4 in FZQ-F after exposure to the LC_50_ dose of thiacloprid (Figure 5C,D). After 3 h of exposure, MpPxd2 and MpPxd4 were not significantly upregulated. Exposure for 6, 12, 24, and 48 h significantly increased the expression of both MpPxd2 and MpPxd4. Compared with the controls, the induction of MpPxd2 in FZQ-F adults reached a maximum (5.8-fold) at 24 h and then decreased at 48 h after treatment with 76.8 mg/L thiacloprid (Figure 5C). Meanwhile, the induction of MpPxd4 in FZQ-F adults reached a maximum (4.9-fold) at 12 h and then also decreased at 48 h (Figure 5C). Nevertheless, no significant induction of MpPxd2 or MpPxd4 was identified in FZQ-F adults after 72 h of treatment with thiacloprid at 76.8 mg/L when compared with the controls (Figure 5C,D).

### 3.8. Effect of Knockdown of the MpPxd2 and MpPxd4 Genes on the Sensitivity of M. persicae to Neonicotinoids

**Figure 6:**
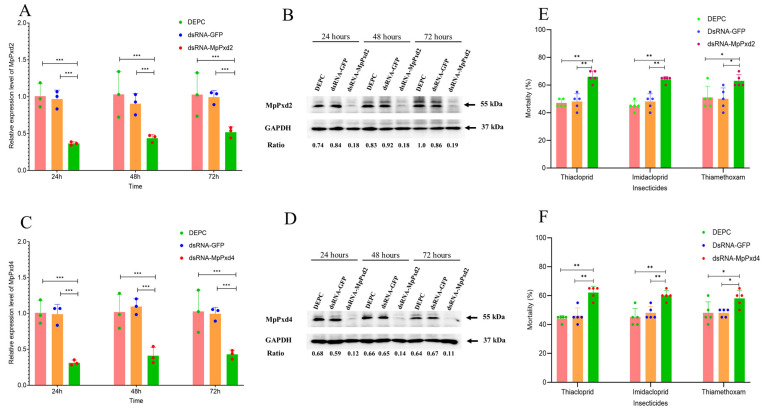
Knockdown of MpPxd2 and MpPxd4 enhances the sensitivity of resistant aphids to imidacloprid, thiacloprid, and thiamethoxam: (**A**) qPCR analysis of MpPxd2 relative expression levels; (**B**) Western blot analysis of MpPxd2 expression levels under various treatments, with relative protein levels normalized to the loading control (GAPDH); (**C**) qPCR analysis of MpPxd4 relative expression levels; (**D**) Western blot analysis of MpPxd4 expression levels under various treatments, with relative protein levels normalized to the loading control (GAPDH); (**E**) percentage mortality at 72 h of adult aphids fed with dsRNA-MpPxd2 after treatment with thiacloprid (76.8 mg/L), imidacloprid (116.48 mg/L), and thiamethoxam (33.2 mg/L); and (**F**) (%) mortality at 72 h of dsRNA-MpPxd4 feeding adult aphids after treatment with thiacloprid (76.8 mg/L), imidacloprid (116.48 mg/L), and thiamethoxam (33.2 mg/L). The significant differences are marked by asterisks: * at the 0.05 level; ** at the 0.01 level; *** at the 0.001 level.

“To evaluate the role of MpPxd2 and MpPxd4 in FZQ-F’s resistance to neonicotinoids, we used RNA interference to reduce their expression and then tested the toxicity of imidacloprid, thiacloprid, and thiamethoxam. After 72 h of feeding 4th-instar FZQ-F larvae with dsRNA, the expression of MpPxd2 and MpPxd4 was 0.36- and 0.38-fold lower than in the control group with dsGFP (Figure 6A,C).” Western blot analysis confirmed that knockdown caused the decrease in the expression of MpPxd2 and MpPxd4 (Figure 6B,D). The mortality rates of the dsMpPxd2 treatments with thiacloprid, imidacloprid, and thiamethoxam were 66%, 64%, and 63%, which were significantly higher than those of the controls at the LC_50_ dose of these neonicotinoids (Figure 6E). The mortality rates of the dsMpPxd4 treatments with imidacloprid and thiacloprid were 62%, 60%, and 58%, which were significantly higher than those of the controls at the LC_50_ dose of these neonicotinoids (Figure 6F).

### 3.9. Effect of Knockdown of the MpPxd2 and MpPxd4 Genes on Adult Longevity and Offspring Production at Different Temperatures of FZQ-F

**Figure 7:**
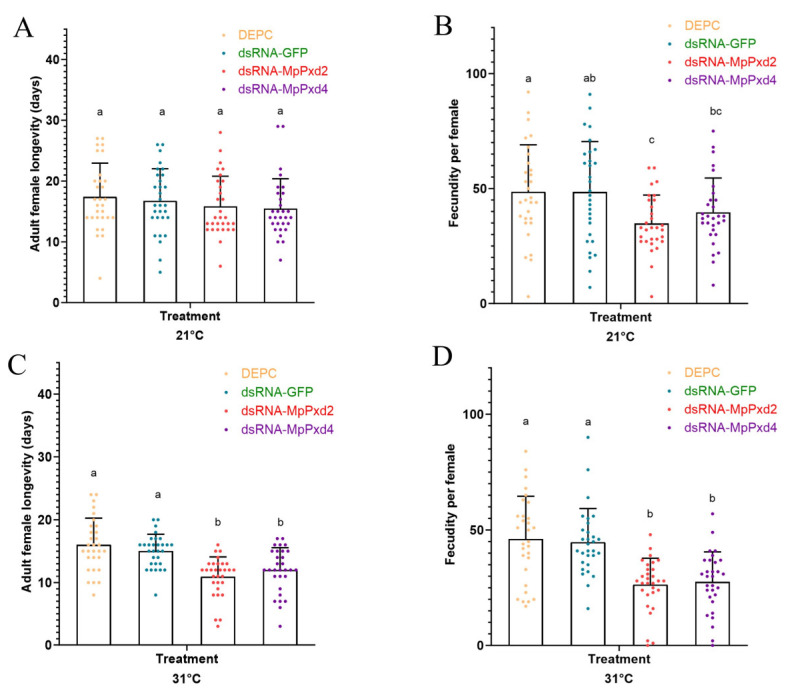
Effects of knockdown of MpPxd2 and MpPxd4 on adult female longevity and fecundity per female at 21 °C and 31 °C. (**A**) Adult female longevity at 21 °C after different treaments; (**B**) Fecundity per female at 21 °C after different treaments; (**C**) Adult female longevity at 31 °C after different treaments; (**D**) Fecundity per female at 31 °C after different treaments. The bars marked with lowercase letters (a–c) indicate significant differences as determined by one-way ANOVA followed by Tukey’s multiple comparison test (*p* < 0.05).

The adult longevity of *M. persicae* and fecundity per female at 21 °C and 31 °C were investigated after the RNAi of MpPxd2 and MpPxd4. At 21 °C, there were no significant differences in adult longevity between the RNAi of MpPxd2- and MpPxd4-treated aphids and the DEPC aphids (Figure 7A). Aphids treated with dsRNA-MpPxd4 had the lowest fecundity (34.8 offspring), significantly lower than the other three treatments (Figure 7B). However, at 31 °C, the longevity of apterous adults and the fecundity per female treated with dsRNA-MpPxd2 or dsRNA-MpPxd4 were significantly shorter than when treated with DEPC or dsRNA-GFP (Figure 7C,D).

## 4. Discussion

Neonicotinoids have been extensively employed in the management of *M. persicae* in the cabbage fields of Fuzhou. We previously reported that a field population (SEF-R) collected in 2023 evolved different levels of resistance to thiacloprid (29-fold), imidacloprid (106.9-fold), and thiamethoxam (12.9-fold). In this research, the FZQ-F developed higher resistance to thiacloprid (44.1-fold) and thiamethoxam (17.5-fold) than the SEF-R [4]. The two populations were both collected from the Fuzhou district, and the resistance levels to the two neonicotinoids improved only one year ago. We detected 74.49% R81T (an arginine to threonine substitution at the 81 site) in nAChRs and the overexpression of some metabolic genes in SEF-R, indicating the existence of the two major mechanisms responsible for neonicotinoid resistance [4]. However, pest ability to overcome other negative effects caused by large increases in insecticide dosages, such as oxidative stress, have received less attention.

Neonicotinoids are formulated to selectively target insect nAChRs, and high resistance means decreased selectivity. Neonicotinoids also exhibit genotoxicity in human and animal cells, which was ignored in the target pests. Neonicotinoids can result in the production of ROS, leading to toxic effects on non-target organisms [38,39]. In this research, the doses of thiacloprid used to induce ROS in FZQ-F adults were 11.77 mg/L and 28.62 mg/L, respectively. We saw that both doses of thiacloprid were able to greatly increase the production of ROS in the intestines of FZQ-F aphids. We did not use the susceptible strain FFJ-S as the control, because the adult females of FFJ-S did not survive after exposure to the two doses. Thiacloprid induction led to a higher production of ROS in FZQ-F after a 24 h exposure to both treatment doses. The earthworm *Eisenia fetida*, when exposed to imidacloprid at a dose of 0.66 mg/kg, also showed a similar result, increasing the production of ROS after 24 h of exposure [40]. A study by Parny et al. [41] discovered that thiacloprid (1, 10 μmol/L) made human monocyte-derived macrophages (hMDMs) better at making ROS. Ge et al. [39] reported that the zebrafish *Danio rerio* produced a lot more ROS when they were exposed to high concentrations of imidacloprid at 1.25 and 5 mg/L. Similarly, zebrafish livers exposed to increasing doses of nitenpyram—another neonicotinoid—also produced more ROS [42]. The production of ROS in the intestines of FZQ-Fdecreased after 72 h of exposure to thiacloprid, indicating a reduction in oxidative stress.

Hydrogen peroxide is the most stable ROS with the longest half-life, and different ROS are changed into H_2_O_2_ inside cells. The amount of H_2_O_2_ can provide much information about oxidative stress. Previous research has shown that insects and other arthropods exposed to low levels of thiacloprid can produce excessive amounts of H_2_O_2_, which can cause oxidative damage [23]. We saw a significant rise in H_2_O_2_ levels in FZQ-F adults before they were exposed to LC_10_ and LC_25_ doses of thiacloprid, suggesting that the insecticide caused oxidative stress. In 2024, Rymuszka and Sieroslawska discovered in *C. carpio* that thiacloprid induced much more H_2_O_2_ when it came into contact with cells from both primary and continuous cultures [23]. Thiacloprid exposure at levels of 11.77 mg/L and 28.62 mg/L caused a dose-dependent increase in H_2_O_2_ levels in FZQ-Fover 3 to 24 h. The highest H_2_O_2_ content occurred 24 h after exposure, indicating when the greatest oxidative stress took place. These results might indicate the improved ability to scavenge H_2_O_2_ in aphids.

To protect insects from oxidative stress, antioxidative systems must neutralize H_2_O_2_ and other reactive oxygen species generated by environmental stress. As an important factor, POD is involved in the scavenging of H_2_O_2_ by oxidizing substrates such as guaiacol and ascorbate. In this research, there was no significant difference in POD activity between 11.77 and 28.62 mg/L thiacloprid, indicating that the induction of POD was not associated with the treatment concentration. However, only on the second day did we observe a significant induction of POD activities at 11.77 and 28.62 mg/L thiacloprid; the activities of POD enzymes and the of H_2_O_2_ contents all increased, indicating that POD plays a crucial role in protecting the FQZ-F aphids from oxidative stress at the beginning of exposure. The POD activity significantly increased at lower imidacloprid concentrations over short-term exposures in some exposed animals. In contrast, previous studies indicated that the POD activity of *E. fetida* showed a significant increase at 0.66 mg/kg imidacloprid concentration over the entire duration of exposure [40]; however, only the first day showed a significant induction of POD activity at 2 and 4 mg/kg imidacloprid. *Eisenia fetida* needed POD to protect it from antioxidant stress at lower imidacloprid concentrations and for short periods of time [40]; this is not consistent with our results.

Pxd plays an important role in the insect innate immune system. The Pxd genes in *M. persicae* have a unique, stable structure that is typical of heme peroxidase genes; they have two conserved cysteine residues at the N-terminus and C-terminus that can each form a disulfide bond. Other insects, such as *Anopheles sinensis* and *A. aegypti*, also exhibit this conserved structure. Regarding molecular weight, the Pxd gene in *M. persicae* ranges from 61.94 to 153.4 kDa [13]. In Diptera species, such as *A. sinensis*, *Anopheles gambiae*, and *Cu. pipiens*, the molecular weight of Pxd mostly falls between 61.6 and 186.6 kDa. However, the number of Pxd genes shows significant variations in different insects. Our study found that there were thirty-one Pxd genes in *M. persicae*, compared to twenty-four in *B. mori* and eight in *M. domestica*. For other members of the Diptera order, there are 20 Pxd genes in *A. sinensis*, 10 Pxd genes in *D. melanogaster*, 12 Pxd genes in *Cu. pipiens*, 14 Pxd genes in *A. aegypti*, and 18 Pxd genes in *A. gambiae* [13]. Therefore, the peach aphid has an amplified number of Pxd genes compared to the aforementioned insects. Different insect species have varying numbers of Pxd genes, suggesting that Pxd regulates immunomodulators, influenced by the distinct environmental conditions to which these insects must adapt. Invertebrates, lacking adaptive immunity, rely instead on an innate immunity. In this context, the cell adhesion protein Pxd, which responds to foreign bodies in invertebrates, appears to be a crucial component of their cellular defense response [43]. However, the correlation between the increased number of Pxd genes and the strength of innate immunity is not yet clear.

Currently, there is no well-defined classification system for insect heme peroxidases (Pxds) based on their genetic structure. Before our study, no polygenetic analysis was conducted in Hemipterans. The analysis of phylogenetic trees is the most effective approach to understanding the relationships among insect Pxds. Our research explored the phylogenetic relationships of Pxd genes in three insect species: *M. persicae*, *A. pisum*, and *B. tabaci*. We classified these genes into five distinct groups. Groups I and II contained most Pxd genes in the three hemipterans. Groups V contained only two genes from *M. persicae* and *A. pisum* (MpPxd12 and AcPxd7). The remaining four groups each encompass Pxd genes derived from the three species. The phylogenetic tree indicated that Groups I, II, and III are ancestral, with Group IV and V possibly evolving later. The Pxd genes within Groups I and II in *M. persicae* and *A. pisum* showed a closer relationship to one another. The number of Pxd genes in aphids is significantly higher than in whiteflies, suggesting that Pxd genes in aphids may possess a broader range of functions. In [44], a phylogenetic analysis was conducted of Pxd genes in five Diptera species: *A. sinensis*, *D. melanogaster*, *A. gambiae*, *A. aegypti*, and *Cu. quinquefasciatus*. They sorted these genes into 10 groups, suggesting that *A. sinensis* and *A. gambiae* have a close evolutionary link.

Comparative transcriptome profiling revealed candidate MpPxd genes linked to *M. persicae* thiacloprid resistance. We found an overexpression of MpPxd2 and MpPxd4 in FZQ-F compared to FFJ-S (Figure 4A). Polygenetic analysis and gene notation classified MpPxd2 as a chorion peroxidase-like gene and MpPxd4 as an mLt-7-like peroxidase, both belonging to Group IV, and thiacloprid significantly induced the two MpPxd gene transcripts at the tested dosages for 24 h (Figure 4B). The 76.8 mg/L thiacloprid-exposed groups had significantly increased H_2_O_2_ contents, POD activities, and mRNA transcript levels of the two MpPxds during the treatment period before 48 h. There were no significant differences in the POD activities or expression levels of the two MpPxds between the FZQ-F groups that had not been treated with thiacloprid and those that had been exposed to 76.8 mg/L of it for 72 h. MpPxd2 and MpPxd4 were the first reported heme POD genes in insects exposed to thiacloprid. There have been fewer studies on the effects of insecticides on insect Pxd gene expression. TRX genes, encoding a stress-response protein that belongs to the thioredoxin peroxidase system, were found to be upregulated in some insect pests—such as *B. mori*, the fall armyworm *Spodoptera frugiperda*, and the crustacean *Daphnia magna*—when exposed to the insecticide chlorantraniliprole [45,46,47]. However, the Trx-1 in *A. mellifera* exhibited no effect under imidacloprid treatments [48]. Trx genes were also not upregulated in FZQ-F exposed to thiacloprid (unpublished data).

Previous research has shown that insect peroxidases do not directly interact with insecticides [49,50]. However, these proteins are antioxidants that protect against oxidative stress caused by pesticide exposure [51,52]. In our research, RNAi-mediated knockdown of MpPxd2 and MpPxd4 significantly decreased their expression. After silencing MpPxd2 and MpPxd4, FZQ-F adults became more sensitive to neonicotinoids, with a higher mortality rate than the control group. Researchers have already shown that insects have developed a way to deal with xenobiotic stress. This method involves reactive oxygen species (ROS), the cap’n’collar isoform-C (CncC), and its heterodimeric partner—muscle aponeurosis fibromatosis (Maf). These components function as transcription factors, leading to the overexpression of enzymes and transporters that contribute to insecticide resistance [9,53]. We did not investigate these related pathways and genes, which may responsible for thiacloprid resistance in *M. persicae*.

Research has shown that various environmental factors, such as temperature extremes, can lead to oxidative stress [54]. After the knockdown of MpPxd2 and MpPxd4, we compared adult longevity and fecundity per female with those of untreated aphids. After the RNAi of the two genes, we found that adult longevity is temperature-dependent, indicating an important role in temperature tolerance. A similar result has been found previously, where the mutant of the Cysu gene—a heme peroxidase—led to shortened lifespan in *D. melanogaster* [55]. George et al. [56] also observed a slight decrease in *C. elegans* longevity when they knocked down skpo-1 (ShkT-containing peroxidase). Otherwise, thioredoxin (Trx) is vital for neutralizing harmful oxidants. Researchers have linked Trx2 to resilience against oxidative stress in *D. melanogaster* [57]. Temperature extremes, UV light, physical damage, and microorganisms such as *E. coli* and the entomopathogenic fungus *Metarhizium anisopliae* significantly induced HaTrx2 in the cotton bollworm *Helicoverpa armigera* [58]. However, this study found that the knockdown of the two MpPxds led to lower fecundity than the control, indicating other functions of the two Pxds. MpPxd2 is a chorion peroxidase-like gene, and MpPxd4 is an MLT-7-like peroxidase. In *A. aegypti*, chorion peroxidase is needed for the chorion to harden, because it speeds up the process of protein crosslinking by creating dityrosine; it is also resistant to denaturing conditions and may contribute to H_2_O_2_ formation during chorion hardening in *A. aegypti* eggs [59]. In the cuticle collagen of *C. elegans*, peroxidase (MLT-7) and NADPH dual oxidase (BLI-3) work together to help reactive oxygen species (ROS) break down covalent bonds [60,61]. Thus, it is highly important and advantageous to conduct further research on the protein structures, functions, and specific active sites of the MpPxds in *M. persicae*. This will aid in the discovery of targeted pest control strategies that minimize impacts on non-target organisms.

## 5. Conclusions

In conclusion, the field population of *M. persicae* (FZQ-F) in Fuzhou still exhibited high resistance to neonicotinoids. Thiacloprid can significantly induce the generation of ROS, including H_2_O_2_, in FZQ-F adults. Peroxidase activity was also significantly increased to protect *M. persicae* from oxidative stress. Two genes—MpPxd2 and MpPxd4, the heme peroxidases—were significantly upregulated by thiacloprid exposure. Function analyses by means of RNAi revealed the protective function of MpPxd2 and MpPxd4 in response to neonicotinoids, and possibly other environmental stresses such as extreme temperatures. Furthermore, the two MpPxds possess additional functions that are necessary for aphid development, which require further study.

## Figures and Tables

**Table 1:** Cross-resistance of FFJ-S and FZQ-F strains of GPA to three neonicotinoids.

Insecticides	Strains	Slope (SE)	LC_10_ (95%CI) Mg/L	LC_25_ (95%CI) Mg/L	LC_50_ (95%CI) Mg/L	χ^2^	RF ^a^
Thiacloprid	FFJ-S	3.65 (0.32)	0.77 (0.63–0.9)	1.13 (0.99–1.27)	1.89 (1.74–2.06)	3.91 (df = 3)	
	FZQ-F	1.57 (0.14)	11.77 (7.8–15.98)	28.62 (21.97–35.42)	76.8 (63.89–93.13)	3.04 (df = 4)	44.1
Imidacloprid	FFJ-S	2.47 (0.2)	0.31 (0.23–0.38)	0.54 (0.45–0.63)	1.09 (0.97–1.22)	2.12 (df = 4)	
	FZQ-F	1.96 (0.18)	21.13 (14.46–27.83)	43.1 (33.63–52.35)	116.48 (97.95–138.88)	3.12 (df = 3)	93.3
Thiamethoxam	FFJ-S	4.62 (0.42)	1.41 (1.2–1.6)	1.91 (1.71–2.09)	2.57 (2.4–3.74)	4.06 (df = 4)	
	FZQ-F	1.81 (0.18)	9.13 (5.87–12.5)	19.82 (14.88–24.63)	33.2 (27.37–40.49)	1.58 (df = 3)	17.5

^a^, RF (resistance factor) = LC_50_ value of theFZQ-F/LC_50_ value of the FFJ-S.

## Data Availability

Datasets analyzed or generated during the study should be obtained by contacting the corresponding authors.

## Supplementary Materials

The following supporting information can be downloaded at: https://www.mdpi.com/article/10.3390/antiox13121453/s1, Table S1: Primers used in the present study; Table S2: Characteristics of heme peroxidase genes of *Myzus persicae*; Table S3: Information of heme peroxidase genes of *Acyrthosiphon pisum* and *Bemisia tabaci*; Figure S1: Multiple sequence alignment of amino acids of 31 MpPxds family members.
