# Supplementary material for: Function Analysis of Heme Peroxidase Genes, MpPxd2 and MpPxd4, Under Thiacloprid Exposure in the Neonicotinoid-Resistant Myzus persicae (Sulzer)
Source: Antioxidants (Basel). 2024 Nov 27;13(12):1453. doi: 10.3390/antiox13121453 (PMC11673278; doi:10.3390/antiox13121453)
Supplement: Supplementary file 1 [file antioxidants-13-01453-s001.zip › antioxidants-3295552-supplementary.pdf]

Supplementary Table S1. Primers used in the present study.

Gene ID	Primer Name	Sequence (5'-3')	Product Length (bp)	Description
836110	Actin-F	GGTGTCTCACACACAGTGCC	222	Real-time PCR primer, forward
	Actin-R	CGGCGGTGGTGGTGAAGCTG		Real-time PCR primer, reverse
LOC111034111	MpPxd4-F	CCGACGATTCTCCAGACTCAA	119	Real-time PCR primer, forward
	MpPxd4-R	CGGGACGGAACAACATTCTTT		Real-time PCR primer, reverse
LOC111032943	MpPxd2-F	TGGCTATTACGCATTCCGATTA	84	Real-time PCR primer, forward
	MpPxd2-R	TGGCTCAGAAAGACCTTAACAT		Real-time PCR primer, reverse
	MpPxd2-RNAiF1	CCATGAAACATATCGCGTTG	364	The first-step PCR primer of dsRNA synthesis, forward
	MpPxd2-RNAiR1	AGTTCTAAGGCGCAGTGCAT		The first-step PCR primer of dsRNA synthesis, reverse
	MpPxd2-RNAiF2	TAATACGACTCACTATAGGGAGA	410	The second-step PCR primer of dsRNA synthesis, forward
	MpPxd2-RNAiR2	CCATGAAACATATCGCGTTG		The second-step PCR primer of dsRNA synthesis, reverse
	MpPxd2-RNAiR2	TAATACGACTCACTATAGGGAGA		The second-step PCR primer of dsRNA synthesis, reverse
	MpPxd4-RNAiR1	AGTTCTAAGGCGCAGTGCAT		The first-step PCR primer of dsRNA synthesis, reverse
LOC111034111	MpPxd4-RNAiF1	TCAGAGAGGTCGTGATGTCG	302	The first-step PCR primer of dsRNA synthesis, forward
	MpPxd4-RNAiR1	AATAGCTTCCCGGTTGACCT		The first-step PCR primer of dsRNA synthesis, reverse
	MpPxd4-RNAiF2	TAATACGACTCACTATAGGGAGA	348	The second-step PCR primer of dsRNA synthesis, forward
	MpPxd4-RNAiR2	TAATACGACTCACTATAGGGAGA		The second-step PCR primer of dsRNA synthesis, reverse
AAB02576.1	GFP-RNAiR1	TCAGAGAGGTCGTGATGTCG		The first-step PCR primer of dsRNA synthesis, reverse
	GFP-RNAiR2	AATAGCTTCCCGGTTGACCT		The second-step PCR primer of dsRNA synthesis, reverse

Table S2. Characteristics of heme peroxidase genes of *Myzus persicae.*

Gene Symbol	Gene ID	Protein ID	Starting Point	End Position	Length of CDS	Number of Exons	Coding Protein Characteristics					
							Amino Acid Length	Molecular Weight (MW)/ (kDa)	Isoelectric Point (PI)	Secondary Structure Characteristics		
										α-Helix (%)	β-Sheet (%)	Random Coil (%)
MpPxd33	LOC111030621	XP_022165910.1	11368	25464	4169	18	1150	127.62	5.57	21.65	18	60.35
MpPxd14	LOC111041243	XP_022181156.1	204585	213063	4350	20	1316	149.12	6.99	25.68	14.74	59.57
MpPxd13	LOC111026692	XP_022160514.1	68818	84868	2430	13	769	86.42	6.61	26.27	18.99	54.75
MpPxd24	LOC111037163	XP_022175244.1	30347	41497	2603	15	726	81.92	6.31	30.03	14.05	55.92
MpPxd34	LOC111037168	XP_022175249.1	43383	57898	2469	15	731	82.42	6.3	29.27	15.87	54.86
MpPxd15	LOC111035817	XP_022173288.1	226656	236862	2973	12	906	101.45	6.96	32.34	12.14	55.52
MpPxd12	LOC111038432	XP_022177219.1	111619	159461	3234	13	838	92.67	5.84	28.76	15.63	55.61
MpPxd27	LOC111034120	XP_022170860.1	150823	164617	2261	13	624	70.54	4.89	22.12	19.07	58.81
MpPxd3	LOC111034115	XP_022170848.1	120561	131823	2218	13	624	71	8.19	20.67	19.87	59.46
MpPxd4	LOC111034111	XP_022170841.1	133232	148016	2742	12	624	70.55	5.61	16.35	22.12	61.54
MpPxd28	LOC111032950	XP_022169140.1	31054	38045	2343	12	691	78.29	6.61	20.26	23.01	56.73
MpPxd19	LOC111032951	XP_022169141.1	16111	25342	2176	14	651	73.9	5.69	21.04	21.97	56.99
MpPxd17	LOC111032948	XP_022169138.1	40536	48183	2484	13	670	77.12	5.53	24.48	19.85	55.67
MpPxd18	LOC111032940	XP_022169124.1	64254	70870	2613	14	675	76.67	5.51	20.44	18.67	60.89
MpPxd2	LOC111032943	XP_022169127.1	49026	57652	2692	15	688	77.95	6.08	23.26	17.73	59.01
MpPxd29	LOC111039011	XP_022177978.1	95714	104151	2483	13	658	75	5.66	18.69	23.4	57.9
MpPxd1	LOC111031232	XP_022166789.1	792515	799213	2164	12	636	72.65	6.4	27.99	17.77	54.25
MpPxd11	LOC111034102	XP_022170828.1	25534	70086	3080	13	654	72.71	7.86	25.08	14.98	59.94
MpPxd30	LOC111027943	XP_022162140.1	76672	81733	2205	13	659	75	6.34	24.43	16.08	59.48
MpPxd25	LOC111027942	XP_022162139.1	88066	103906	4329	26	1351	153.4	8.14	26.5	13.84	59.66
MpPxd20	LOC111033629	XP_022170133.1	486792	493003	2413	13	673	77.51	6.92	27.79	15.9	56.32
MpPxd9	LOC111032921	XP_022169097.1	82645	115940	2850	13	694	78.56	5.75	16.43	16.43	67.15
MpPxd26	LOC111034613	XP_022171594.1	263726	275994	2325	11	774	88.98	6.2	24.29	21.83	53.88
MpPxd8	LOC111034612	XP_022171593.1	248905	253267	2540	11	750	86.28	5.78	26.13	20.53	53.33
MpPxd16	LOC111034617	XP_022171598.1	224640	230699	2571	11	757	87.42	6.03	29.85	22.06	48.08
MpPxd7	LOC111030564	XP_022165804.1	101	3901	1667	7	538	61.94	7.21	28.07	22.30	49.63
MpPxd23	LOC111034614	XP_022171595.1	213248	218188	2525	11	744	85.21	5.62	26.34	18.55	55.11
MpPxd6	LOC111041820	XP_022181949.1	737763	744824	2713	11	759	87.8	8.47	25.69	19.89	54.41
MpPxd21	LOC111029875	XP_022164792.1	286750	314177	2502	11	742	85.05	8.11	27.90	20.08	52.02
MpPxd5	LOC111042342	XP_022182610.1	34566	43035	2545	11	733	84.13	6.23	27.97	21.28	50.75
MpPxd22	LOC111029784	XP_022164640.1	197090	201958	2535	10	740	83.60	8	23.51	20.95	55.54

Table S3. Information of heme peroxidase genes of *Acyrthosiphon pisum* and *Bemisia tabaci.*

Gene Symbol	Protein ID	Gene Symbol	Protein ID
AcPxd1	XP_001944466.1	AcPxd31	XP_016663746.2
AcPxd2	XP_001944512.2	AcPxd32	XP_016664411.1
AcPxd3	XP_001944613.1	AcPxd33	XP_016665023.2
AcPxd4	XP_001946575.2	AcPxd34	XP_029340948.1
AcPxd5	XP_001946672.2	AcPxd35	XP_029341002.1
AcPxd6	XP_001947415.2	AcPxd36	XP_029341003.1
AcPxd7	XP_001947809.2	AcPxd37	XP_029341297.1
AcPxd8	XP_001948315.1	AcPxd38	XP_029341367.1
AcPxd9	XP_001948369.2	AcPxd39	XP_029341375.1
AcPxd10	XP_001949287.2	AcPxd40	XP_029341434.1
AcPxd11	XP_001950289.2	AcPxd41	XP_029341470.1
AcPxd12	XP_003241493.3	AcPxd42	XP_029341799.1
AcPxd13	XP_003241916.2	AcPxd43	XP_029342676.1
AcPxd14	XP_003247028.1	AcPxd44	XP_029345881.1
AcPxd15	XP_003248730.1	AcPxd45	XP_029348684.1
AcPxd16	XP_008180110.2	AcPxd46	XP_029348713.1
AcPxd17	XP_008181566.1	BtPxd1	XP_018900724.1
AcPxd18	XP_008181994.2	BtPxd2	XP_018900796.1
AcPxd19	XP_008183733.1	BtPxd3	XP_018900823.1
AcPxd20	XP_008184911.1	BtPxd4	XP_018902871.1
AcPxd21	XP_008186761.2	BtPxd5	XP_018904211.1
AcPxd22	XP_008188186.2	BtPxd6	XP_018906184.1
AcPxd23	XP_008189676.2	BtPxd7	XP_018906644.1
AcPxd24	XP_016656256.1	BtPxd8	XP_018907162.1
AcPxd25	XP_016657105.1	BtPxd9	XP_018909479.1
AcPxd26	XP_016657151.1	BtPxd10	XP_018909483.1
AcPxd27	XP_016662601.1	BtPxd11	XP_018909743.1
AcPxd28	XP_016662602.1	BtPxd12	XP_018911279.1
AcPxd29	XP_016662957.1	BtPxd13	XP_018916648.1
AcPxd30	XP_016663586.1		

Note: AcPxd: heme peroxidase of *Acyrthosiphon pisum*; BtPxd: heme peroxidase of *Bemisia tabaci*.

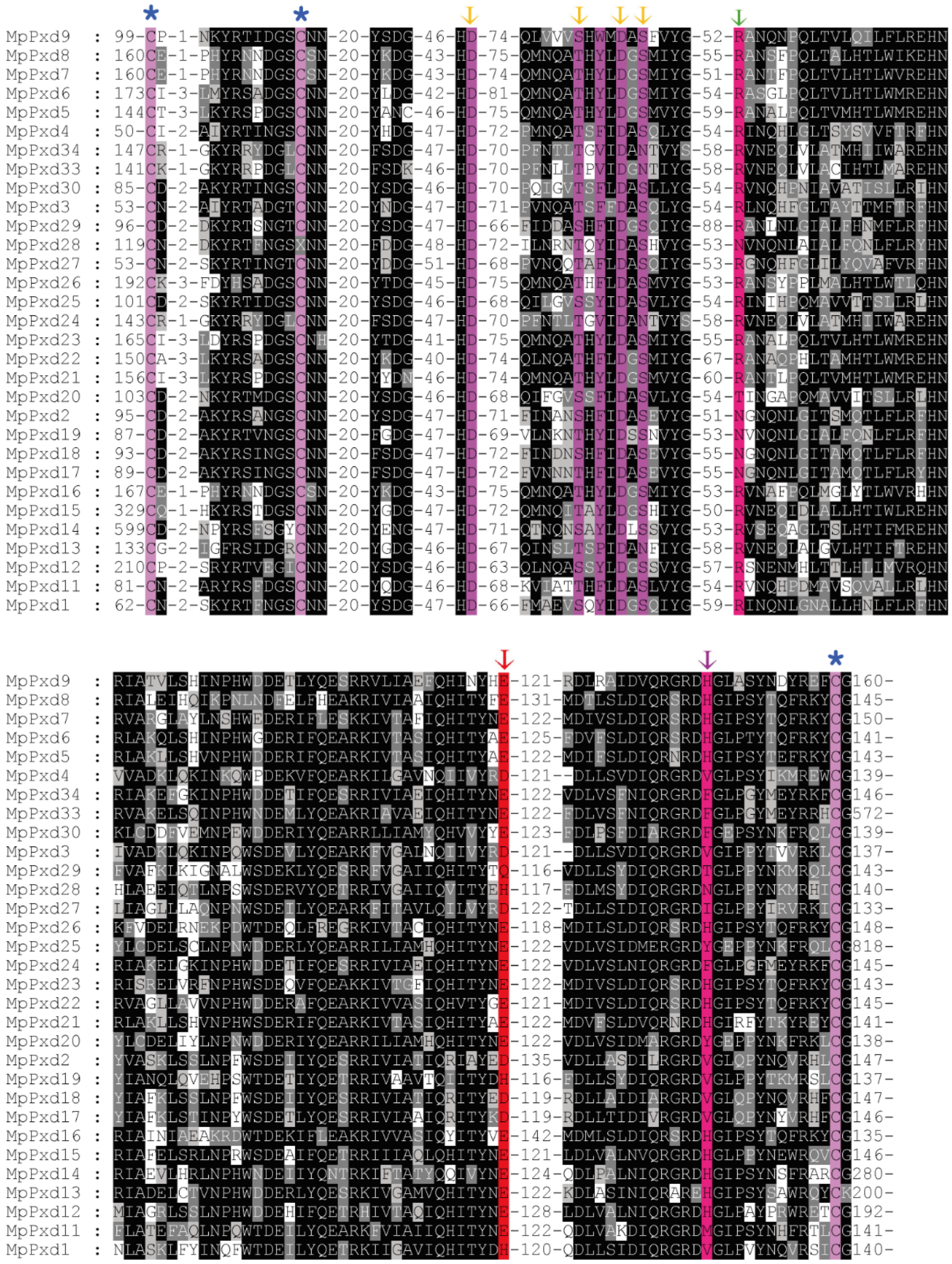

Figure S1. Multiple sequence alignment of amino acids of 31 MpPxds family members. Asterisks indicate cysteine, the four yellow arrows indicate Ca2+-binding sites, the green arrow indicates the transition state stabilizer site, the red arrow indicates the heme-binding site, and the purple arrow indicates the iron-ion-binding site.
